# A One-Year Retrospective Observational Study of an Occupational Medicine Outpatient Clinic in a City Hospital

**DOI:** 10.5334/aogh.4978

**Published:** 2025-10-27

**Authors:** Yusuf Samir Hasanli

**Affiliations:** 1 Ankara Etlik City Hospital, Occupational Diseases Unit, Varlik Neighborhood, Halil Sezai Erkut Street, 06170 Yenimahalle, Ankara, Türkiye

**Keywords:** occupational diseases, personal protective equipment, employment duration, occupational health, workplace

## Abstract

*Background:* Occupational diseases (ODs) remain a major public health concern. Despite advances in occupational health (OH), many workers remain at risk due to insufficient preventive measures and limited access to specialized care.

*Objective:* This study aimed to evaluate the sociodemographic and occupational characteristics of patients attending an ODs outpatient clinic in Türkiye and to assess associated risk factors.

*Methods:* We retrospectively analyzed data from 326 patients in a descriptive observational study. Variables included age, sex, work duration, income, education, personal protective equipment (PPE) use, and diagnosis.

*Findings:* The mean age was 48.1 years, and 89.3% of participants were male. ODs were diagnosed in 42.3% of patients, with pneumoconiosis being the most common (34.1%). Longer employment duration significantly increased risk (OR: 1.06; 95% CI: 1.03–1.09; *p* < 0.001). Income was also significant: both below- (OR: 2.79; 95% CI: 1.20–6.49; *p* = 0.017) and above-minimum-wage earners (OR: 2.52; 95% CI: 1.00–6.37; *p* = 0.050) had a higher risk. Most participants reported dust (79.4%) and ergonomic exposures (77.6%). Consistent mask use was 12.0% and hearing protection 7.7%. PPE use was insufficient in smaller workplaces but relatively higher in larger ones. Women began working later than men (26.2 vs. 17.3 years; *p* < 0.001). Most participants (85.9%) had social security coverage, yet 16% worked after retirement.

*Discussion:* Improving OH practices, particularly in small enterprises where PPE use is inadequate, expanding worker training and workplace inspections, and ensuring access to OH services for self-employed individuals are critical for effective prevention of ODs. Additionally, addressing socioeconomic factors such as income disparities may further reduce occupational disease risk.

## Introduction

Occupational diseases (ODs) affect individual health and significantly impact workforce productivity and economic sustainability. In the context of ongoing industrial and technological development, the importance of their diagnosis and management has increased. Occupational medicine specialists play a key role in early detection, accurate evaluation, and the development of preventive strategies.

The International Labor Organization (ILO) defines ODs as “diseases that have a direct causal relationship with a specific occupation or occupational exposure” [1]. However, obtaining reliable data on the global prevalence and incidence of ODs is challenging due to inconsistent and often incomplete reporting systems. Harrington *et al*. estimate that 4–12 new occupational disease cases occur annually per 1,000 workers [2]. In Türkiye, official figures remain much lower. According to 2023 Social Security Institution (SSI) data, only 945 workers received compensation after an OD diagnosis, defined as ≥10% loss in earning capacity [3]. This number only covers insurance-based cases, excluding clinically diagnosed conditions that do not meet legal criteria or go unreported.

Work-related diseases remain a serious public health issue globally. The ILO reports approximately 160 million new non-fatal cases each year [4]. In the United Kingdom, 1.7 million new or existing work-related health cases were reported in 2023/24, of which 46% were stress-related, 32% musculoskeletal disorders, and 21% other ODs [5]. In Germany, the Social Accident Insurance reported a >60% drop in suspected OD cases in 2023, with confirmed cases falling by 63.5% to 72,747 [6].

Informal employment further reduces the visibility of ODs. ILO data show that in 2024, 27.7% of employment in Türkiye was informal [7]. Consequently, this large workforce is excluded from OD diagnosis, monitoring, and reporting, resulting in reported case numbers that fall well below estimates and highlighting systemic deficiencies.

The global increase in OD-related deaths is striking. Between 2014 and 2019, such deaths rose from 2.3 million to 2.9 million, a 26% increase [8]. A joint WHO (World Health Organization) and ILO report estimated 1.9 million deaths in 2016 due to occupational risk factors (ORFs), 81% of which were disease-related, and 19% due to injuries [9]. The most mortality-linked ORFs are ≥55-hour work weeks, followed by exposure to particulate matter, gases, fumes, and occupational injuries. The highest mortality burden is associated with chronic obstructive pulmonary disease (COPD), stroke, and ischemic heart disease, and this burden is disproportionately higher in WHO regions such as Africa, Southeast Asia, and the Western Pacific, especially among men and older individuals [10].

This study aims to present a one-year clinical experience in an ODs outpatient clinic at a city hospital, thereby elucidating the profile of ODs in Türkiye. Real-world data from ODs clinics in Turkey remain extremely limited. Awareness and reporting of ODs remain insufficient both nationally and in similar settings. By analyzing the sociodemographic, occupational, and exposure profiles of patients attending the clinic, this study seeks to contribute to local health policies and OH practices. It also represents a valuable field study that may guide international comparisons aimed at improving the management of ODs in developing countries.

## Methods

### Study design

This study has a retrospective, observational, and descriptive design. The STROBE (strengthening the reporting of observational studies in epidemiology) checklist was followed for reporting.

### Patient population

All patients who presented to the ODs outpatient clinic of Ankara Etlik City Hospital between January 2024 and January 2025 were included in the study (*n* = 326). Those who did not complete the diagnostic process were retained in the dataset and classified as “discontinued cases.” For individuals with multiple visits, only the first presentation was analyzed. Accordingly, no exclusion criteria were applied. In Türkiye, there are approximately 60 OD specialists, and the OD subspecialty has existed for only about 10 years, making it a relatively new field. The ODs outpatient clinic at Ankara Etlik City Hospital is staffed by a single specialist, and all patients presenting during the study period are included. Diagnosed cases are recorded in the hospital information system, formally referred to the SSI for official notification, and additionally reported to the Ministry of Health via monthly email. Patients categorized as “With Occupational Disease” were newly diagnosed at our hospital by the author and consulting physicians, as all patients presented specifically for evaluation of ODs.

### Data collection process

The file number and visit date for each outpatient were recorded in an SPSS dataset by the researcher. After ethical approval, medical records were retrospectively reviewed in May-June 2025 via the hospital information management system (HIMS) using these file numbers. Data included patient histories, complaints, occupational histories, test results, diagnoses, and clinical decisions. A single OD specialist familiar with clinical workflows collected and evaluated the data. Due to the absence of a second specialist, double data checking was not performed. However, as data were directly recorded in the system and transferred to SPSS, potential entry errors are considered minimal. Although this limits data reliability, it likely does not affect the study’s overall validity.

### Variables and definitions

The main variables evaluated in this study were as follows: age, sex, marital status, education level, income level (IL), social security status, type of admission (referred, scheduled, unscheduled), presenting complaints, retirement status, occupation, diagnosis of ODs, most frequently diagnosed ODs, comorbidities, ORFs, PPE use, occupational accidents, unhealthy habits, and body mass index (BMI).

IL was categorized into three groups based on self-reports, using the minimum wage (MW) defined by the Turkish Statistical Institute as reference: below MW, at MW, and above MW. Occupations were grouped according to the International Standard Classification of Occupations (ISCO-08). BMI was calculated from height and weight measured in the clinic and classified according to WHO criteria.

ORFs were assessed via face-to-face clinical interviews at presentation. No questionnaires or standardized scales were used; instead, exposures were determined from patient self-reports, sector-specific occupational knowledge, and physician clinical experience. Each occupational risk category—for example, physical hazards such as noise, extreme temperatures, vibration, and radiation—was classified as “positive” if at least one relevant risk factor was present. Chemical, ergonomic, psychosocial, dust, and biological risks were evaluated similarly.

PPE use was generally classified as users or non-users. The use of masks, safety glasses, and earplugs was further divided into four subgroups: non-users despite need (e.g., miners without masks), non-users without the requirement (e.g., office workers without dust masks), irregular users, and regular users. This classification was made to facilitate basic occupational safety (OS) evaluations and allow for more detailed comparisons based on workplace size (WS). WS was categorized into four groups by employee count: self-employed, 2–10, 11–50, and 50+ employee workplaces.

### Ethical approval

Ethical approval was obtained from the Ankara Etlik City Hospital Ethics Committee (No: AEŞH-BADEK1-2025-093; 07 May 2025). Informed consent was obtained from all participants. The study was conducted in accordance with the Declaration of Helsinki and data protection regulations, with all patient data fully anonymized prior to analysis.

### Statistical analysis

Data were analyzed using IBM SPSS Statistics 27.0. Continuous variables were summarized with mean, median, minimum, maximum, and 95% confidence interval (CI). Categorical variables were presented as counts and percentages. Student’s t-test and Chi-square tests assessed the effects of independent variables on dependent ones. The Chi-square was specifically used for mask, goggles, and earplug use, reflecting OH culture and PPE adherence; other equipment usage was analyzed descriptively. Multivariate binary logistic regression identified factors associated with OD status, including work duration, smoking (pack-years), gender, age at job start, IL, marital status, education, and WS. Statistical significance was set at *p* < 0.05. As this descriptive study covered the entire one-year patient population, no power analysis was performed. Despite this limitation, analyses were appropriately conducted.

## Results

The mean age of the 326 patients was 48.1 years, with a significant difference between males and females (39.0 vs 49.2 years, *p* < 0.001). Patients with ODs were younger than those without (46.2 vs 50.4 years, *p* = 0.007). Mean employment duration differed significantly between women and men (9.1 vs 25.9 years, *p* < 0.001). Those with ODs had shorter employment than those without (20.3 vs 27.8 years, *p* < 0.001). Among 227 current and former smokers, mean pack-years differed between OD and non-OD patients (23.2 vs 29.1, *p* = 0.031). All 80 post-retirement patients were male, with a mean time since retirement of 10.2 years, showing no significant difference between OD and non-OD patients. Mean age at starting work was higher in women than in men (26.2 vs 17.3 years, *p* < 0.001). Age at starting work did not differ significantly by OD status (18.8 vs 17.7 years, *p* = 0.076), though it was close to significance. For more detailed results, see Table 1.

**Table 1:** Distribution of continuous variables of the study population.

VARIABLE	NUMBER (*N*)	MEAN (95% CI)	MEDIAN	MIN-MAX	*P*-VALUE
Age (years)	Total *n* = 326	48.1 (46.7–49.6)	47.5	18–80	
	“Women (*n* = 35)	39.0 (35.5–42.4)	40.0	20–69	<0.001
	Men (*n* = 291)	49.2 (47.7–50.8)	49.0	18–80	
	*With OD (*n* = 138)	46.2 (43.8–48.6)	44.0	19–76	0.007
	Without OD (*n* = 168)	50.4 (48.5–52.3)	51.0	23–80	
Work Duration (years)	Total *n* = 326	24.1 (22.6–25.6)	25.0	1–60	
	“Women (*n* = 35)	9.1 (6.8–11.5)	7.0	1–26	<0.001
	Men (*n* = 291)	25.9 (24.4–27.4)	27.0	1–60	
	With OD (*n* = 138)	20.3 (18.0–22.6)	18.0	1–60	<0.001
	Without OD (*n* = 168)	27.8 (25.9–29.7)	30.0	1–57	
**Smoking (pack-years)	Total *n* = 227	26.8 (24.0–29.4)	22.0	1–100	
	With OD (*n* = 98)	23.2 (19.4–27.0)	20.0	1–100	0.031
	Without OD (*n* = 119)	29.1 (25.3–32.9)	25.0	1–100	
Body Mass Index (kg/m²)	Total *n* = 326	26.8 (26.4–27.3)	26.6	17.5–41.5	
	“Women (*n* = 35)	26.8 (25.1–28.4)	26.0	20–37	0.941
	Men (*n* = 291)	26.9 (26.4–27.3)	26.7	17.5–41.5	
	With OD (*n* = 138)	26.5 (25.8–27.1)	26.6	18.1–37.7	0.124
	Without OD (*n* = 168)	27.2 (26.5–27.9)	26.7	17.5–41.5	
***Post-Retirement Duration (years)	Total *n* = 80	10.2 (8.4–12.1)	9.0	1–36	
	“With OD (*n* = 30)	12.1 (8.4–15.7)	10.0	1–36	0.151
	“Without OD (*n* = 47)	9.3 (7.2–11.4)	8.0	1–32	
Age at Workforce Entry (years)	Total *n* = 326	18.3 (17.7–18.8)	18.0	8–50	
	“Women (*n* = 35)	26.2 (23.3–29.1)	25.0	15–50	<0.001
	Men (*n* = 291)	17.3 (16.9–17.8)	17.0	8–39	
	With OD (*n* = 138)	18.8 (17.8–19.9)	18.0	8–50	0.076
	Without OD (*n* = 168)	17.7 (17.1–18.4)	18.0	10–39	

*OD: Occupational Disease. Patients who did not complete the OD evaluation process (*n* = 20) were excluded from the analysis. Analyses were conducted only between patients diagnosed with an OD and those not diagnosed.

**Total number of current and former smokers: 227. After excluding patients who discontinued the diagnostic process, the total number of smokers in the groups with and without OD diagnosis was 217 (with OD/without OD = 98/119).

***The number of patients presenting after retirement was 80, all male. No further analysis was conducted. The total number of individuals with and without an OD diagnosis was 77 (three patients discontinued the diagnostic process). The distribution was 30 with and 47 without an OD diagnosis.

“Estimates for very small subgroups (*n* < 50) should be interpreted with caution due to limited sample size.

Of the 326 patients assessed, 89.3% were male. The majority of participants were married (90.8%). In terms of education, the largest group were primary school graduates (52.5%). OD was diagnosed in 42.3% of patients, while 51.5% were not diagnosed with OD following evaluation, and 6.1% withdrew from the process. Among patients diagnosed with OD, the most common was pneumoconiosis (34.1%). Of the 326 patients, 35.0% had no comorbidities; the most common were chronic lung diseases (20.5%) and cardiovascular diseases (13.5%). Among patients, 48.2% were current smokers, 30.4% never smoked, and 21.4% had quit. Respiratory symptoms were the most common presenting complaint, reported by 59.5%. The largest occupational group by ISCO-08 major categories was “Craft and Related Trade Workers” at 52.8%, followed by “Elementary Occupations” at 18.4%, and “Plant and Machine Operators and Assemblers” at 13.2%. The majority of patients (62%) were referred to the OD unit by other hospital departments; only 5.2% were referred by workplace physicians. Regarding social security status, 85.9% were insured employees. Of the 326 patients, 14.7% were retired and not working, while 16% were retired but still working. Exposure to workplace risk factors was common: 86.8% faced at least one physical risk, and 66.9% at least one chemical risk. Dust exposure affected 79.4%, ergonomic risks 77.6%, psychosocial risks 12.0%, and biological risks 6.4%. Regarding mask use, 44.5% of those required to wear a mask never did, while only 12.0% used one regularly. Among those needing ear protection, 49.4% never used it, and 7.7% used it regularly. Among those needing safety glasses, 22.4% never used them, while 31.6% used them regularly. Of participants, 6.1% were self-employed. Additionally, 21.8% worked in facilities with 2–10 employees, 29.4% in those with 11–50 employees, and 42.6% in facilities with more than 50 employees. For more detailed results, see Supplementary Table 1.

Multivariable logistic regression analysis showed the model was statistically significant overall (Omnibus Test, which evaluates whether the model as a whole predicts the outcome better than a null model, *p* < 0.001). The Hosmer–Lemeshow test (assessing goodness-of-fit between observed and predicted values) indicated adequate fit (*p* = 0.241). Longer employment duration was significantly associated with increased occupational disease risk (OR: 1.06, 95% CI: 1.03–1.09; *p* < 0.001). No significant associations were found between smoking (pack-years) or age at workforce entry and OD. Male sex was linked to lower risk compared to females (OR: 0.26, 95% CI: 0.05–1.43, *p* = 0.119), not statistically significant. No significant overall association was found between WS and OD (*p* = 0.129), though risk tended to increase in subcategories. Compared to the self-employed, workers in facilities with 2–10 employees had 3.15-fold higher risk (OR: 3.15, 95% CI: 0.75–13.17; *p* = 0.117), with 11–50 employees 2.09-fold higher (OR: 2.09, 95% CI: 0.99–4.43; *p* = 0.055), and over 50 employees 2.04-fold higher (OR: 2.04, 95% CI: 0.91–4.60; *p* = 0.084). These approached but did not reach significance. IL had a significant overall effect on OD risk (*p* = 0.050). Those earning above minimum wage had 2.52 times higher risk (OR: 2.52, 95% CI: 1.00–6.37; *p* = 0.050), and those earning below minimum wage had 2.79 times higher risk (OR: 2.79, 95% CI: 1.20–6.49; *p* = 0.017). No significant association was found between education level and OD (*p* = 0.623). Primary school graduates were the reference group. Risk was 1.26 times higher for middle school education (OR: 1.26, 95% CI: 0.25–6.28; *p* = 0.779) and 29% lower for high school graduates (OR: 0.71, 95% CI: 0.11–4.39; *p* = 0.697). Marital status was significantly associated with OD (*p* = 0.005). Single individuals had 84% lower risk compared to married individuals (OR: 0.16, 95% CI: 0.04–0.58; *p* = 0.006). For more detailed results, see Table 2.

**Table 2:** Odds ratios of factors associated with occupational disease risk according to multivariable logistic regression analysis.

INDEPENDENT VARIABLE		B	S.E.	WALD χ²	OR [%95 CI]	*P*-VALUE
Work Duration (years)		0.06	0.02	12.51	1.06 [1.03–1.09]	<0.001
Smoking (pack-years)		0.01	0.01	1.07	1.01 [0.99–1.03]	0.300
BMI (kg/m²)		0.00	0.04	0.00	1.00 [0.93–1.07]	0.990
Age at Workforce Entry (years)		0.02	0.04	0.27	1.02 [0.95–1.10]	0.599
Gender (Female/Male)		−1.36	0.88	2.43	0.26 [0.05–1.42]	0.119
Workplace Size				5.67		0.129
Reference Category: Self-Employed	2–10 Workers	1.15	0.73	2.46	3.15 [0.75–13.17]	0.117
	11–50 Workers	0.74	0.38	3.68	2.09 [0.99–4.43]	0.055
	> 50 Workers	0.72	0.41	2.97	2.04 [0.91–4.60]	0.084
Income Level				6.00		0.050
Reference Category: Minimum Wage	Above Minimum Wage	0.93	0.47	3.83	2.52 [1.00–6.37]	0.050
	Below Minimum Wage	1.03	0.43	5.65	2.79 [1.20–6.49]	0.017
Education Level				1.76		0.623
Reference Category: Primary School*	Middle School	0.23	0.82	0.07	1.26 [0.25–6.28]	0.779
	High School	−0.34	0.93	0.14	0.71 [0.11–4.39]	0.708
	University	−0.09	0.82	0.01	0.91 [0.18–4.56]	0.905
Marital Status (Married/Single)		−1.88	0.68	7.59	0.16 [0.04–0.58]	0.006

B: Beta coefficient, S.E.: Standard Error, Wald: Wald statistic (tests each variable’s contribution to the model), OR: Odds Ratio, CI: Confidence Interval.

*Note*: The overall model significance was determined by the Omnibus Test (*p* < 0.001) and the Hosmer–Lemeshow Test (*p* = 0.241).

*Primary school was selected as the reference category. The group with no formal education (*n* = 6) was not used as a reference due to the small sample size.

**Figure 1:**
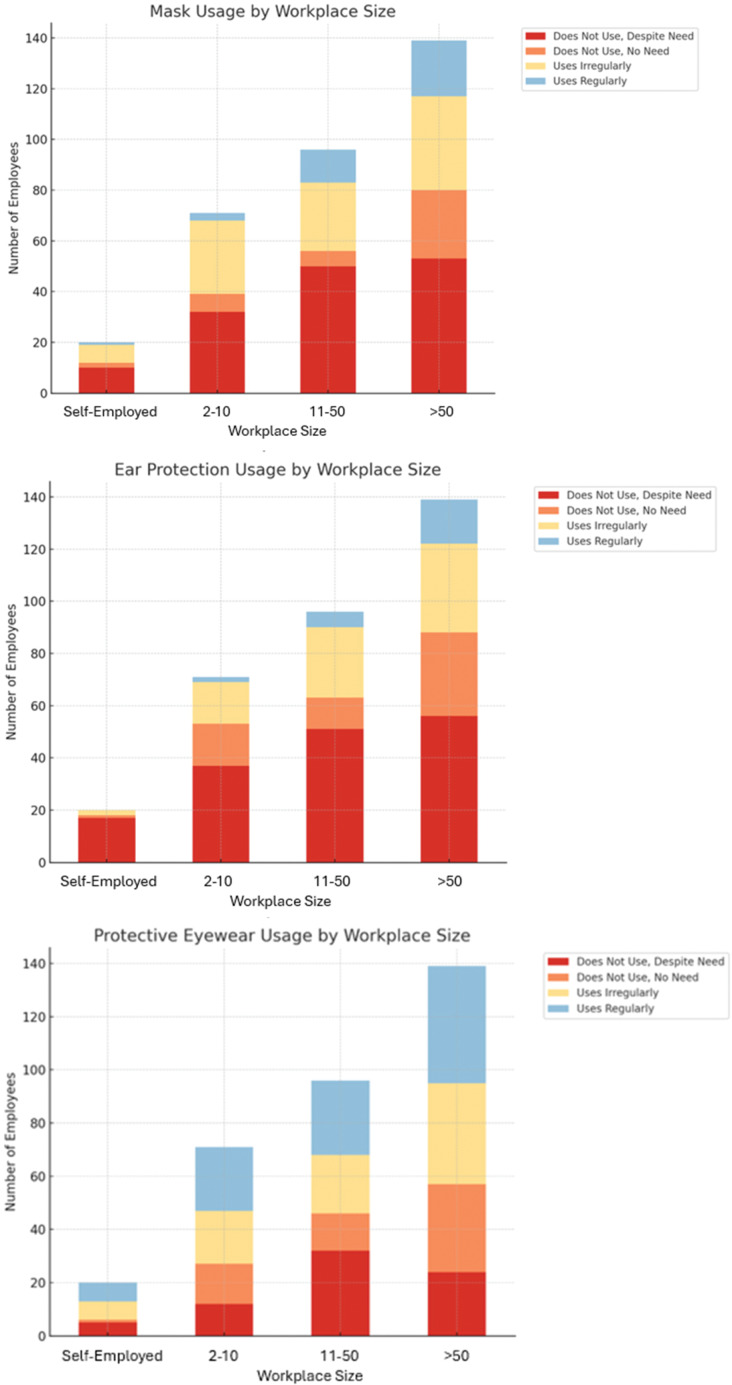
Distribution of mask usage status, ear protection usage status, and protective eyewear usage status by workplace size.

Analysis of mask use by WS showed a significant association (Chi-square, *p* = 0.013). Among the self-employed, 50.0% did not use a mask despite needing one, compared to 45.1% in workplaces with 2–10 employees. Regular mask use increased with WS, reaching 15.8% in workplaces with over 50 employees. Overall, 44.5% did not wear a mask when required, while 12.0% used one regularly. Ear protection use by WS also showed a significant association (Chi-square, *p* = 0.005). Among the self-employed, 85.0% did not use ear protection despite needing it, compared to 52.1% in workplaces with 2–10 employees. Although the use slightly increased with WS, regular use remained low in facilities with over 50 employees (12.2%). Overall, 49.4% did not use ear protection when required. No significant difference was found in safety glasses use by WS (Chi-square, *p* = 0.122). Regular use was slightly higher among the self-employed (35%) compared to 29–34% in others. Overall, 31.6% used them regularly, while 22.4% did not use glasses despite needing them. For more detailed results, see Supplementary Table 2 and Figure 1.

## Discussion

ODs remain a significant global public health concern. Although awareness has increased in Turkey and worldwide, efforts in diagnosis, reporting, and prevention remain insufficient. This study analyzes the sociodemographic and occupational characteristics of patients attending a city hospital’s OD clinic, providing up-to-date field data from Türkiye. The findings indicate that disease risk is associated with employment duration, gender, income, and IL, with PPE use and exposure intensity also playing key roles. These results offer valuable insights for local healthcare practice and can inform international OH policies.

In our study, the average ages of all patients attending the clinic within one year and those diagnosed with OD were consistent with the literature. For instance, our findings align with U.S. survey data indicating that work-related health problems are most commonly reported among individuals aged 45–54 [11]. Additionally, longer duration of employment was found to be significantly associated with an increased risk of OD, with each additional year of work corresponding to a 6% rise in risk. This finding underscores the critical role of cumulative exposure in disease development and highlights the need for early preventive occupational health (OH) policies worldwide. The Helsinki Health Study reported a significantly higher risk of disability retirement due to musculoskeletal disorders among individuals engaged in heavy physical work for 8–10 years [12].

In our study, the average duration of employment among individuals diagnosed with OD (20.3 years) was significantly shorter than that of those without a diagnosis (27.8 years), indicating that total work duration alone is insufficient for OD development, and that exposure intensity and protective measures must also be considered. The literature shows that high exposure intensity can rapidly lead to severe ODs; for example, the risk of pneumoconiosis is 4.6 times higher with long-term exposure compared to short-term exposure (RR: 4.59; 95% CI: 2.41–8.74; *p* < 0.01), and each additional 5 years of exposure increases the risk by 26% (RR: 1.26; 95% CI: 1.15–1.39; *p* < 0.01) [13]. In this study, the observation that women (9.1 years) had shorter working durations than men (25.9 years) may explain their earlier presentation to the clinic, and could also be influenced by factors such as exposure intensity, health awareness, or work/life conditions. However, the limited number of previous studies supporting this finding restricts the ability to compare our results with existing research.

Smoking is more prevalent among individuals with lower education levels and blue-collar workers, and it adversely affects multiple organs [14]. Therefore, smoking cessation interventions should be promptly implemented following the diagnosis of pneumoconiosis or other occupational lung diseases [15]. Interestingly, in our study, individuals diagnosed with OD had a lower mean pack-year history (23.2) compared to those without a diagnosis (29.1). This inverse relationship may be attributable to smoking cessation after diagnosis or shorter smoking duration among younger patients; however, no studies in the literature directly support this observation.

The significantly higher workforce entry age among women compared to men (26.2 vs. 17.3 years) suggests the impact of marriage and childcare responsibilities. UN Women and ILO’s global analysis reports that marriage and parenthood often reduce women’s labor force participation but increase it for men [16]. This may partly explain why women enter the workforce at a later age and, as a result, experience a shorter duration of occupational exposure compared to men.

The concentration of men in high-risk sectors contributes to higher rates of work-related accidents and OD mortality. According to 2023 ILO data, the global fatality rate is 51.4 per 100,000 for men and 17.2 per 100,000 for women [17]. In addition to these gender-related occupational risks, educational level plays an important role in workers’ vulnerability to occupational hazards. Globally, 72% of workers have education below their job requirements, indicating that low education is a widespread workforce issue [18]. Similarly, 63.2% of the patients in our study had low education, making them more susceptible to occupational risks. Raising the education level is crucial for the prevention of work-related accidents and ODs.

Although the incidence of pneumoconiosis has declined since 2015, it remains a significant OH concern, with 527,500 cases and over 60,000 new cases reported in 2017 [19]. Similarly, pneumoconiosis was identified as the most common OD in our cohort. The majority of cases were among craft workers (52.8%) and those in elementary-level jobs (18.4%), which is markedly higher than the corresponding rates in the European Union (EU). In the EU, the most common occupations are professionals (21%) and technicians (17%), while craft and elementary-level jobs account for 12% and 8%, respectively [20]. While comparisons with EU data are illustrative, our clinic-based sample may not represent national rates. Furthermore, the predominance of primary school graduates among our patients supports the notion that the Turkish workforce has a low level of education, which may increase the tendency to engage in hazardous occupations.

In 2020, 46.9% of the global population was covered by social protection, compared to 83.9% in Europe and Central Asia [21]. The rate observed in our study (85.9%) exceeds the global average, although it may not fully capture informal employment. Additionally, 16% of patients continue to work after retirement, which is higher than the 10.2% observed in the EU [22]. Low pension levels in Türkiye may contribute to this situation. Strengthening social security and better managing post-retirement employment are critical for workforce protection.

Women face different and unique occupational risks compared to men, and this should be considered in OH policies [23]. A population-based survey found that women are about 30% more likely than men to report repetitive tasks and high-paced work. Additionally, women reported higher exposure to disinfectants, hair dyes, and textile dust compared to men [24]. Extensive research shows that women face poorer working conditions and higher job insecurity than men. A systematic review covering 1999–2010 found that women report worse psychosocial environments and poorer physical and mental health compared to men [25]. In our study, men had a lower risk of OD than women (OR: 0.26, 95% CI: 0.05–1.43, *p* = 0.119), indicating women’s risk was about 3.85 times higher (1/0.26 ≈ 3.85). This trend was not statistically significant, but it is consistent with gender-based risk disparities reported in the literature. Some associations did not reach statistical significance and are presented as exploratory findings for hypothesis generation. International studies suggest women workers may face higher risks due to biological differences, PPE designed primarily for male ergonomics, and workplace gender discrimination [26]. Developing gender-based OH policies is a critical issue that needs to be addressed globally.

One of the interesting and discussion-worthy findings in our study was IL; individuals earning above the minimum wage had a 2.52-fold higher risk of OD, while those earning below had a 2.79-fold higher risk. This U-shaped paradoxical relationship can be explained by the fact that low-income workers often hold riskier and less supervised jobs, whereas high-income workers face longer working hours and heavier workloads. International studies highlight this income-health paradox and emphasize equal access to OH regardless of income. A Dutch study found low socioeconomic workers had 2.7 times higher OD risk than high socioeconomic position (SEP) workers, with musculoskeletal and noise-induced hearing loss more common in low SEP, and stress-related diseases more prevalent in high SEP groups [27].

According to ILOSTAT data, marital status significantly affects the global labor market. Singles face higher unemployment risk, while married individuals experience more underemployment and job underutilization. Combined with gender roles, married women become especially vulnerable in the labor market [28]. In our study, the 84% lower rate of OD among singles compared to married individuals is likely due to their younger age and the longer hours and heavier workloads of married workers driven by family responsibilities. These findings highlight the importance of considering sociodemographic factors in OH policies.

Our study found a strong link between WS and PPE use, especially masks and earplugs. The use of PPE is relatively more consistent in large enterprises, indicating a more established OH and safety culture. Serious gaps exist in OH practices in small businesses, and self-employed workers show low PPE use—issues reported in Europe and developing countries [29]. This highlights the need to expand OH policies to cover all business types.

### Strengths and limitations

A key strength of this study is its reflection of real-world OH data, which allows for multivariate analyses and potential international comparisons. However, several limitations should be acknowledged. The study’s single-center, retrospective chart review design may limit generalizability and is subject to recall bias in patient-reported exposures. The relatively low proportion of female workers may have constrained gender-based analyses. Data entry was performed by a single researcher without double-checking, introducing a potential risk of recording errors. Exposure assessments relied on clinical interviews rather than standardized questionnaires or scales, which may introduce subjectivity and diagnostic or expectation bias. Despite these limitations, the study provides valuable insights into real-world OD patterns in a setting where such data are scarce.

## Conclusion

This study highlights OD as a significant public health issue both nationally and globally. Strengthening diagnosis, registration, and reporting processes is critical for effective disease management and health policy development. OH practices should be widely implemented regardless of workplace size, with a focus on increasing PPE use in small enterprises and among self-employed workers. Targeted training, inspection programs for small businesses, and improved access to OH services for the self-employed are key steps toward international OH goals. These findings may guide the prevention and management of OD in Türkiye and similar countries.

## Data Availability

The data that support the findings of this study are available from the corresponding author upon reasonable request.

## Additional Files

The additional files for this article can be found as follows:

Supplementary Table S1. Key Categorical Characteristics of the Study Group.

Supplementary Table S2. Personal Protective Equipment Usage by Workplace Size and Chi-Square Analysis.
