# Supplementary material for: A One-Year Retrospective Observational Study of an Occupational Medicine Outpatient Clinic in a City Hospital
Source: Ann Glob Health. 2025 Oct 27;91(1):72. doi: 10.5334/aogh.4978 (PMC12577557; doi:10.5334/aogh.4978)
Supplement: Supplementary Table S1. — Key Categorical Characteristics of the Study Group. [file agh-91-1-4978-s1.pdf]

| Supplementary Table 1. Key Categorical Characteristics of the Study Group | | | |
|---|---|---|---|
| Variable | Category | n | % |
| Gender | Women | 35 | 10.7 |
| | Men | 291 | 89.3 |
| Marital Status | Married | 296 | 90.8 |
| | Single | 30 | 9.2 |
| Education Level | No formal education | 6 | 1.8 |
| | Primary School | 171 | 52.5 |
| | Middle School | 29 | 8.9 |
| | High School | 100 | 30.7 |
| | College / University | 20 | 6.1 |
| Occupational Disease Diagnosis Status | With Occupational Disease | 138 | 42.3 |
| | Without Occupational Disease | 168 | 51.5 |
| | Discontinued Evaluation | 20 | 6.1 |
| Diagnosed Occupational Diseases | Pneumoconiosis | 47 | 34.1 |
| | Occupational Asthma/COPD | 31 | 22.5 |
| | Occupational Musculoskeletal Disorders | 21 | 15.2 |
| | Occupational Toxicological Diseases | 12 | 8.7 |
| | Occupational Skin Diseases | 7 | 5.0 |
| | Occupational Hypersensitivity Pneumonitis | 6 | 4.3 |
| | Diffuse Dust-Related Fibrosis | 6 | 4.3 |
| | Hearing Loss | 6 | 4.3 |
| | Malignant Mesothelioma | 1 | 0.7 |
| | Occupational-Related Pulmonary Hypertension | 1 | 0.7 |
| Comorbidities | None | 114 | 35.0 |
| | Chronic Lung Diseases (Asthma, COPD, Bronchiectasis) | 67 | 20.5 |
| | Cardiovascular Diseases (Hypertension, Ischemic Heart Disease) | 44 | 13.5 |
| | Diabetes Mellitus and Metabolic Diseases | 20 | 6.1 |
| | Thyroid Diseases | 17 | 5.2 |
| | Rheumatologic and Autoimmune Diseases | 11 | 3.4 |
| | Gastrointestinal Diseases | 10 | 3.1 |
| | Others | 43 | 13.2 |
| Smoking Status | Current Smoker | 157 | 48.2 |
| | Never Smoked | 99 | 30.4 |
| | Ex-Smoker | 70 | 21.4 |
| Alcohol Use | Yes | 24 | 7.4 |
| | No | 302 | 92.6 |
| Income Level | Below Minimum Wage | 64 | 19.6 |
| | Minimum Wage | 85 | 26.1 |
| | Above Minimum Wage | 177 | 54.3 |
| Presenting Complaints at Admission | Respiratory | 194 | 59.5 |
| | No Complaints | 57 | 19.5 |
| | Musculoskeletal | 31 | 9.5 |
| | Neurological/Neuropsychiatric | 16 | 5.3 |
| | Dermatological | 10 | 3.1 |
| | Hearing-Related Complaints | 6 | 1.8 |
| | Others | 11 | 3.4 |

| | | | |
|---|---|---|---|
| ISCO-08 Codes | 0 - Armed Forces Occupations | 1 | 0.3 |
| | 1 - Managers | 2 | 0.6 |
| | 2 - Professionals | 6 | 1.8 |
| | 3 - Technicians and Associate Professionals | 16 | 4.9 |
| | 4 - Clerical Support Workers | 5 | 1.5 |
| | 5 - Service and Sales Workers | 14 | 4.3 |
| | 6 - Skilled Agricultural, Forestry and Fishery Workers | 7 | 2.1 |
| | 7 - Craft and Related Trades Workers | 172 | 52.8 |
| | 8 - Plant and Machine Operators, and Assemblers | 43 | 13.2 |
| | 9 - Elementary Occupations | 60 | 18.4 |
| Patient Referral Pathways | Referred by Other Hospital Departments | 202 | 62 |
| | Self-Referral | 82 | 25.2 |
| | Referred by Social Security Institution | 19 | 5.8 |
| | Referred by Workplace Physician | 17 | 5.2 |
| | Other Pathways (Labor Court, Other Institutions) | 6 | 1.8 |
| Social Security Status | Employees under Social Security (Dependent Workers) | 280 | 85.9 |
| | Self-Employed Individuals | 20 | 6.1 |
| | Government Employees (Civil Servants) | 13 | 4.0 |
| | Uninsured Individuals | 9 | 2.8 |
| | Special Groups (e.g. Migrants, Military Personnel) | 4 | 1.2 |
| Retirement Status | Not Retired | 226 | 69.3 |
| | Retired, Not Working | 48 | 14.7 |
| | Retired, Still Working | 52 | 16 |
| Work Accident | No | 258 | 79.1 |
| | Once | 52 | 16 |
| | More Than Once | 16 | 4.9 |
| *Exposure to Physical Risks | Yes | 283 | 86.8 |
| | No | 43 | 13.2 |
| * Exposure to Chemical Risks | Yes | 218 | 66.9 |
| | No | 108 | 33.1 |
| * Exposure to Biological Risks | Yes | 21 | 6.4 |
| | No | 305 | 93.6 |
| * Exposure to Dust | Yes | 259 | 79.4 |
| | No | 67 | 20.6 |
| * Exposure to Ergonomic Risks | Yes | 253 | 77.6 |
| | No | 73 | 22.4 |
| * Exposure to Psychosocial Risks | Yes | 39 | 12 |
| | No | 287 | 88 |
| Mask Usage | Does Not Use, Despite Need | 145 | 44.5 |
| | Does Not Use, No Need | 42 | 12.9 |
| | Uses Irregularly | 100 | 30.7 |
| | Uses Regularly | 39 | 12 |
| Protective Earplug Usage | Does Not Use, Despite Need | 161 | 49.4 |
| | Does Not Use, No Need | 61 | 18.7 |
| | Uses Irregularly | 79 | 24.2 |
| | Uses Regularly | 25 | 7.7 |
| Workwear Usage | Does Not Use | 39 | 12 |
| | Uses | 287 | 88 |
| Work Shoe Usage | Does Not Use | 90 | 27.6 |
| | Uses | 236 | 72.4 |

| | | | |
|---|---|---|---|
| Protective Eyewear Usage | Does Not Use, Despite Need | 73 | 22.4 |
| | Does Not Use, No Need | 63 | 19.3 |
| | Uses Irregularly | 87 | 26.7 |
| | Uses Regularly | 103 | 31.6 |
| Hard Hat Usage | Does Not Use | 235 | 72.1 |
| | Uses | 91 | 27.9 |
| Protective Glove Usage | Does Not Use | 129 | 39.6 |
| | Uses | 197 | 60.4 |
| Workplace Size | Self-Employed, Working Alone | 20 | 6.1 |
| | Facility With 2–10 Workers | 71 | 21.8 |
| | Facility With 11–50 Workers | 96 | 29.4 |
| | Facility With More Than 50 Workers | 139 | 42.6 |

n: Number

%: Percentage

COPD: Chronic Obstructive Pulmonary Disease

*Exposure to at least one of the specified risk factors was considered positive. For example, exposure to at least one physical risk factor (such as noise, vibration, hot/cold environments, radiation).
