# Supplementary material for: A One-Year Retrospective Observational Study of an Occupational Medicine Outpatient Clinic in a City Hospital
Source: Ann Glob Health. 2025 Oct 27;91(1):72. doi: 10.5334/aogh.4978 (PMC12577557; doi:10.5334/aogh.4978)
Supplement: Supplementary Table S2. — Personal Protective Equipment Usage by Workplace Size and Chi-Square Analysis. [file agh-91-1-4978-s2.pdf]

Supplementary Table 2. Personal Protective Equipment Usage by Workplace Size and Chi-Square Analysis

Workplace Size	Mask Usage Status Number (n) / Percentage (%)				
	Does Not Use, Despite Need	Does Not Use, No Need	Uses Irregularly	Uses Regularly	Total
Self-Employed	10 (50%)	2 (10%)	7 (35%)	1 (5%)	20 (100%)
2-10 Workers	32 (45.1%)	7 (9.9%)	29 (40.8%)	3 (4.2%)	71 (100%)
11-50 Workers	50 (52.1%)	6 (6.3%)	27 (28.1%)	13 (13.5%)	96 (100%)
>50 Workers	53 (38.1%)	27 (19.4%)	37 (26.6%)	22 (15.8%)	139 (100%)
Total	145 (44.5%)	42 (12.9%)	100 (30.7%)	39 (12.0%)	326 (100%)
	Pearson Chi-Square=20.94, df=9, p=0.013				
	Ear Protection Usage Status Number (n) / Percentage (%)				
	Does Not Use, Despite Need	Does Not Use, No Need	Uses Irregularly	Uses Regularly	Total
Self-Employed	17 (85%)	1 (5%)	2 (10%)	0 (0%)	20 (100%)
2-10 Workers	37 (52.1%)	16 (22.5%)	16 (22.5%)	2 (2.8%)	71 (100%)
11-50 Workers	51 (53.1%)	12 (12.5%)	27 (28.1%)	6 (6.3%)	96 (100%)
>50 Workers	56 (40.3%)	32 (23%)	34 (24.5%)	17 (12.2%)	139 (100%)
Total	161 (49.4%)	61 (18.7%)	79 (24.2%)	25 (7.7%)	326 (100%)
	Pearson Chi-Square=23.86, df=9, p=0.005				
	Protective Eyewear Usage Status Number (n) / Percentage (%)				
	Does Not Use, Despite Need	Does Not Use, No Need	Uses Irregularly	Uses Regularly	Total
Self-Employed	5 (25%)	1 (5%)	7 (35%)	7 (35%)	20 (100%)
2-10 Workers	12 (16.9%)	15 (21.1%)	20 (28.2%)	24 (33.8%)	71 (100%)
11-50 Workers	32 (33.3%)	14 (14.6%)	22 (22.9%)	28 (29.2%)	96 (100%)
>50 Workers	24 (17.3%)	33 (23.7%)	38 (27.3%)	44 (31.7%)	139 (100%)
Total	73 (22.4%)	63 (19.3%)	87 (26.7%)	103 (31.6%)	326 (100%)
	Pearson Chi-Square=14.01, df=9, p=0.122				

df: Degrees of Freedom
